# Highly efficient ultra-broad beam silicon nanophotonic antenna based on near-field phase engineering

**DOI:** 10.1038/s41598-022-23460-x

**Published:** 2022-11-05

**Authors:** Shahrzad Khajavi, Daniele Melati, Pavel Cheben, Jens H. Schmid, Carlos A. Alonso Ramos, Winnie N. Ye

**Affiliations:** 1 Department of Electronics, Carleton University, 1125 Colonel By Drive, Ottawa, ON K1S 5B6 Canada; 2 Centre for Nanoscience and Nanotechnologies, CNRS, Université Paris-Saclay, 10 Bv. Thomas Gobert, 91120 Palaiseau, France; 3 Advanced Electronics and Photonics Research Center, National Research Council Canada, 1200 Montreal Road, Ottawa, ON K1A 0R6 Canada

**Keywords:** Silicon photonics, Integrated optics

## Abstract

Optical antennas are a fundamental element in optical phased arrays (OPA) and free-space optical interconnects. An outstanding challenge in optical antenna design lies in achieving high radiation efficiency, ultra-compact footprint and broad radiation angle simultaneously, as required for dense 2D OPAs with a broad steering range. Here, we demonstrate a fundamentally new concept of a nanophotonic antenna based on near-field phase-engineering. By introducing a specific near-field phase factor in the Fraunhofer transformation, the far-field beam is widened beyond the diffraction limit for a given aperture size. We use transversally interleaved subwavelength grating nanostructures to control the near-field phase. A Bragg reflector is used at the end of the grating to increase both the efficiency and the far-field beam width. The antenna has a compact footprint of 3.1 µm × 1.75 µm and an ultra-broad far-field beam width of 52° and 62° in the longitudinal and transversal direction, respectively, while the radiation efficiency reaches 82% after incorporating a bottom reflector to further improve the directionality. This unprecedented design performance is achieved with a single-etch grating nanostructure in a 300-nm SOI platform.

## Introduction

Optical beam steering is a fundamental functionality at the core of many technologies, including free-space optical communications, 3D imaging and mapping, interconnects, and optical memories^1–3^. Ideally, a beam steering system would be small and lightweight to be mounted on a vehicle or a satellite, or integrated on a handheld device, such as a smartphone^4^. However, state-of-the-art optical beam steering systems typically comprise mechanical assemblies, moving parts and bulk optic components^5^. Optical phased arrays (OPAs) have gained significant interest as a static, non-mechanical beam-steering devices^6–10^. OPAs can be integrated on chip to achieve beam steering by controlling the phase of the light emitted by the antennas forming the array. Optical antennas are the fundamental elements of on-chip arrays^10–13^, with antenna efficiency, aperture size and far-field radiation angle being the key parameters determining the OPA performance. Surface grating couplers have been extensively used in combination with silicon-based planar waveguides for fiber-chip coupling and wafer-scale testing^14–21^. However, for the maximum overlap with optical fiber mode and high coupling efficiency, grating couplers are usually 10–15 µm long. Substantially more compact antenna designs are required for dense OPAs with a wide beam steering range^22–27^. An appealing solution to reduce the antenna dimension while maintaining a high efficiency is to enhance the grating strength by using the 300 nm SOI platform instead of the more common 220 nm one^12,28–30^. We have recently demonstrated an antenna with high diffraction efficiency exceeding 89% and compact footprint of 7.6 µm × 4.5 µm on a 300 nm SOI^31^. While this constitutes an important advance in terms of antenna footprint and efficiency, a remaining outstanding challenge is the antenna’s limited far-field radiation angle, which directly contributes to the definition of the OPA’s steering range.

In this work, we demonstrate, for the first time, a novel design of an ultra-compact silicon grating antenna for off-chip light emission, with an unprecedented performance combining a wide far-field beam width, a very compact footprint, and a high diffraction efficiency. This is achieved by a single-etch grating structure and a Bragg reflector designed on a 300-nm-thick SOI platform. The design methodology is described in the antenna far-field broadening section. The simulation results are presented in the design and simulation results section, and the conclusions are summarized in the discussion and conclusion section.

## Antenna far-field broadening

The far-field distribution generated by an optical antenna is related to the near field by the Fraunhofer transformation^32^:

1 $$U\left(x,y,z\right)\propto {\iint }_{A}^{ }E\left({x}^{\mathrm{^{\prime}}},{y}^{\mathrm{^{\prime}}}\right){e}^{-i\left(\frac{k\left({x}^{\mathrm{^{\prime}}}x+{y}^{\mathrm{^{\prime}}}y\right)}{z}\right)}d{x}^{\mathrm{^{\prime}}}d{y}^{\mathrm{^{\prime}}},$$

where *E(x’,y’)* is the complex amplitude at the aperture A located at *x’y’* plane, and k is the wave vector. For the Fraunhofer approximation to be valid, it is required that D^2^/Zλ <  < 1, where D is the aperture width, Z is the distance between the aperture and the observation point, and λ is the wavelength^33^. This condition is always met by the operating far field conditions of practical OPAs. As described by Eq. (1), the shape and size of the antenna aperture directly affects the far-field distribution. While large beam widths in the far-field can be obtained by using small apertures, reducing the aperture size limits the antenna’s efficiency.

Here, we propose a new strategy to circumvent this fundamental limit leveraging near-field phase engineering^34,35^. Instead of adjusting the antenna aperture, the key idea is to broaden the far-field by judiciously controlling the phase of the complex near-field *E(x’,y’)*. By introducing a specific near-field phase factor in the Fraunhofer transformation, the far-field beam width can be widened without shortening the antenna. This strategy allows us to decouple the minimum antenna length (required to ensure a given scattering efficiency) from the far-field beam width.

The near-field phase could be altered by longitudinally chirping the antenna grating period. However, as we show later in the longitudinally-chirped antenna section, for short antennas with strong radiation strength the chirping effect is ineffective because the antenna comprises only a small number of periods (e.g., 3–5) and their respective contributions to the scattered field are rapidly decreasing along the antenna. If a conventional longitudinal chirp is implemented in such short antennas, the contribution of the last periods to the scattered field is substantially smaller compared to the first period. This constraint fundamentally limits the effectiveness of chirping. Instead of chirping the grating longitudinally, here we use, for the first time, transversally-interleaved chirped nanostructures to control the near-field phase. With transverse chirping, the different chirp periods equally contribute to the scattered field, enabling effective phase engineering in very short gratings (few period) and yielding a substantially broadened far-field radiation compared to the conventional antennas.

## Design and simulation results

### Periodic grating antenna

**Figure 1:**
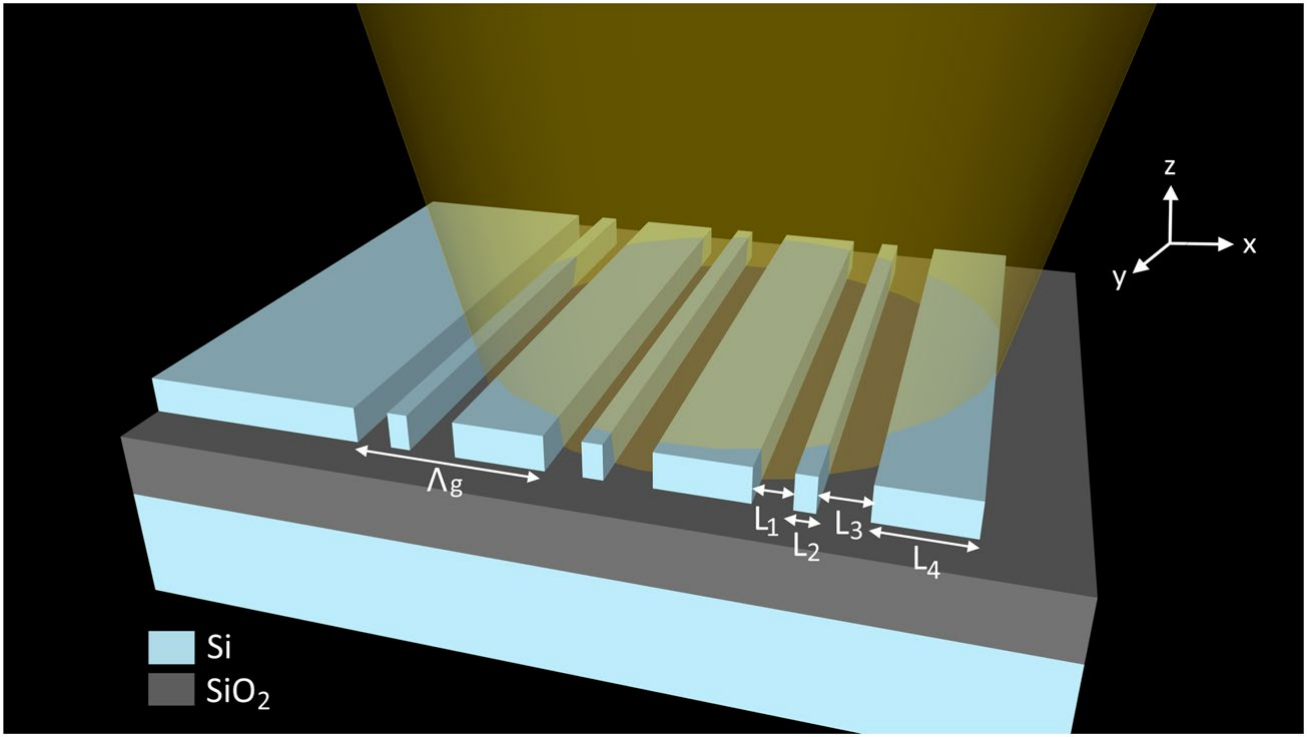
Schematic of the single-etch, un-chirped antenna with grating period Λ_g_ and structural parameters [L_1_, L_2_, L_3_, L_4_]. A transverse electric (TE) waveguide mode is incident from the left.

As a reference case of an antenna with high scattering efficiency^36^, we consider a single-etch, periodic grating in a standard SOI platform with a 300-nm-thick silicon waveguide core, 1-μm buried oxide (BOX) and 2-μm oxide cladding. The grating comprises three periods, each including two fully etched sections with lengths L_1_ and L_3_ and two un-etched sections with length L_2_ and L_4_, as schematically shown in Fig. 1.

**Figure 2:**
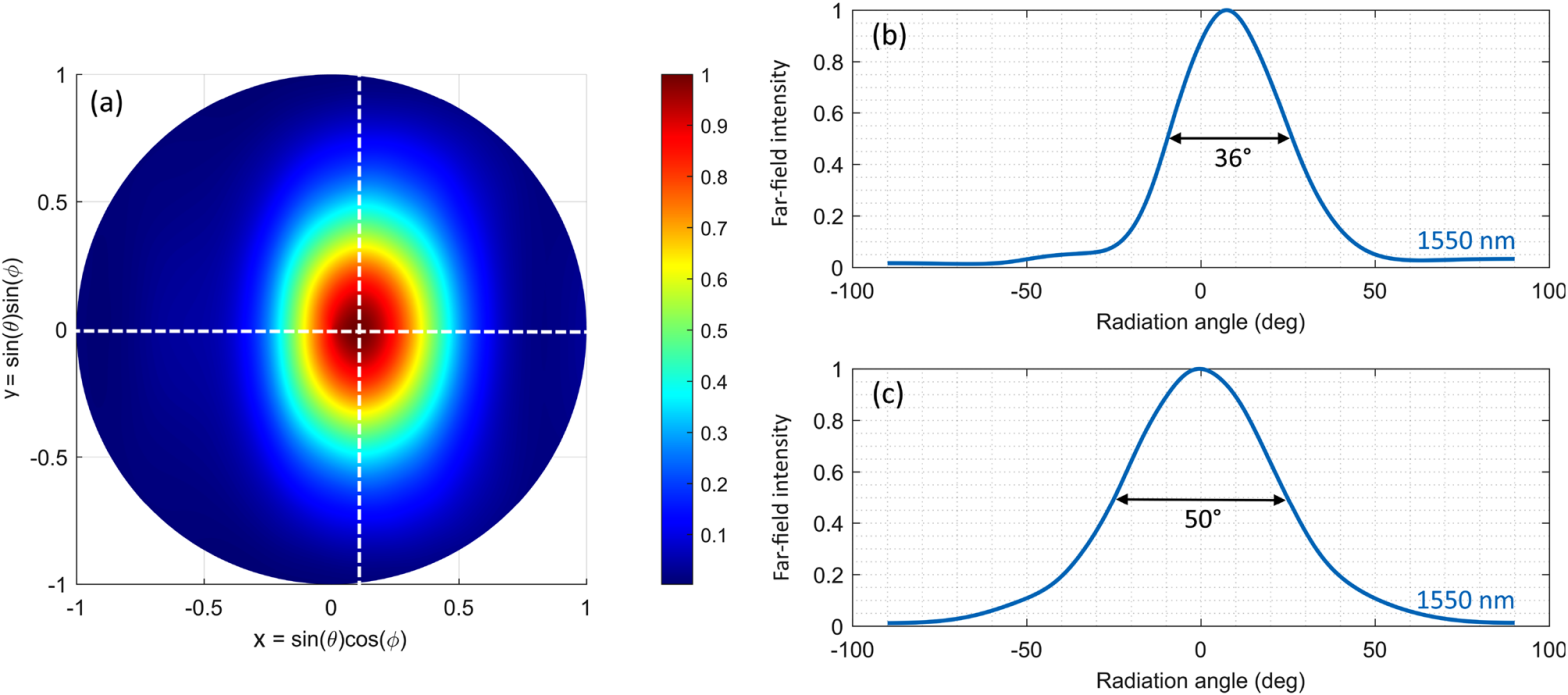
(**a**) The far-field radiation pattern of the reference antenna at a wavelength of 1550 nm. (**b**) Far-field intensity distribution along the x and (**c**) y axis, as marked by the dashed white lines in (**a**).

The four geometrical parameters L_1_–L_4_ are optimized using a genetic algorithm combined with 2D Finite-Difference Time-Domain (FDTD) simulations in order to achieve the maximum upward diffraction efficiency while the diffraction angle is allowed to vary as a free parameter. The detailed optimization procedure can be found in our previous work^31^. The optimized parameters are L_1_ = 109 nm, L_2_ = 48 nm, L_3_ = 189 nm, L_4_ = 387 nm, and Λ_g_ = 733 nm, while the full antenna length is 2.2 μm. Rigorous 3D FDTD simulations are then used to validate the results of the 2D design. The antenna width for this structure is chosen as 1.75 μm, to yield a compact design required for dense antenna arrays. The upward diffraction efficiency at λ = 1550 nm obtained from 2D and 3D simulations is 60% and 55%, respectively, while the grating reflectivity (computed as the fraction of back-reflected power coupled to the counter propagating TE mode of the input waveguide) is − 20 dB (2D) and − 12 dB (3D). It should be noted that shortening the grating further and using two grating periods instead of three would significantly reduce the diffraction efficiency by 11%, while adding a fourth period would only increase efficiency by 2%. Figure 2 shows the far-field radiation pattern for the reference antenna design, where θ and ϕ represent the polar and azimuthal angles, respectively. The peak diffraction angle is 7° from the vertical, and the full width at half maximum (FWHM) of the far-field intensity along the longitudinal (x) and transverse (y) directions is 36° and 50° at 1550 nm, respectively.

### Longitudinally-chirped antenna

In order to evaluate the effect of longitudinal chirping on the far-field beam width, we design an antenna with the structure described in periodic grating antenna section, but considering different periods for the three unit cells. The goal is to engineer the near-field phase profile of the diffracted beam in order to increase the divergence and obtain a broader beam width in the far-field. Simulating a range of grating periods showed that Λ_g1_ < 600 nm results in significant reduction in upward diffraction efficiency. The first cell period was hence designed to provide a large negative diffraction angle of –21°. Cell parameters were optimized as described in the previous section, resulting in L_1_ = 133 nm, L_2_ = 61 nm, L_3_ = 190 nm, and L_4_ = 216 nm (period Λ_g1_ = 600 nm). For the second cell, we use the optimized structural parameters from the reference design of periodic grating antenna section (Λ_g2_ = 733 nm, diffraction angle of 7°). For the last cell period we choose a positive diffraction angle of 9°, which still avoids second-order diffraction. The optimized parameters are L_1_ = 109 nm, L_2_ = 41 nm, L_3_ = 300 nm, and L_4_ = 350 nm, i.e., a cell period of Λ_g3_ = 800 nm. The width of the antenna is 1.75 μm, the same as the reference structure.

3D FDTD was used to simulate the behavior of the longitudinally-chirped grating. We obtained a FWHM of the far-field beam of 38° and 50.2° in the x and y directions, respectively. Diffraction efficiency was reduced to 45% compared to reference design, while maintaining comparable back-reflection (–10 dB). Despite the large chirping from Λ_g1_ = 600 nm to Λ_g3_ = 800 nm, the beam width in the longitudinal direction increased only marginally by 2°, indicating the limited effectiveness of longitudinal chirping to broaden the far-field of short antennas.

### Transversally-interleaved chirped antenna

As described in antenna far-field broadening section, we propose to overcome the ineffectiveness of longitudinal chirping in few-period antennas using transversal chirping.

**Figure 3:**
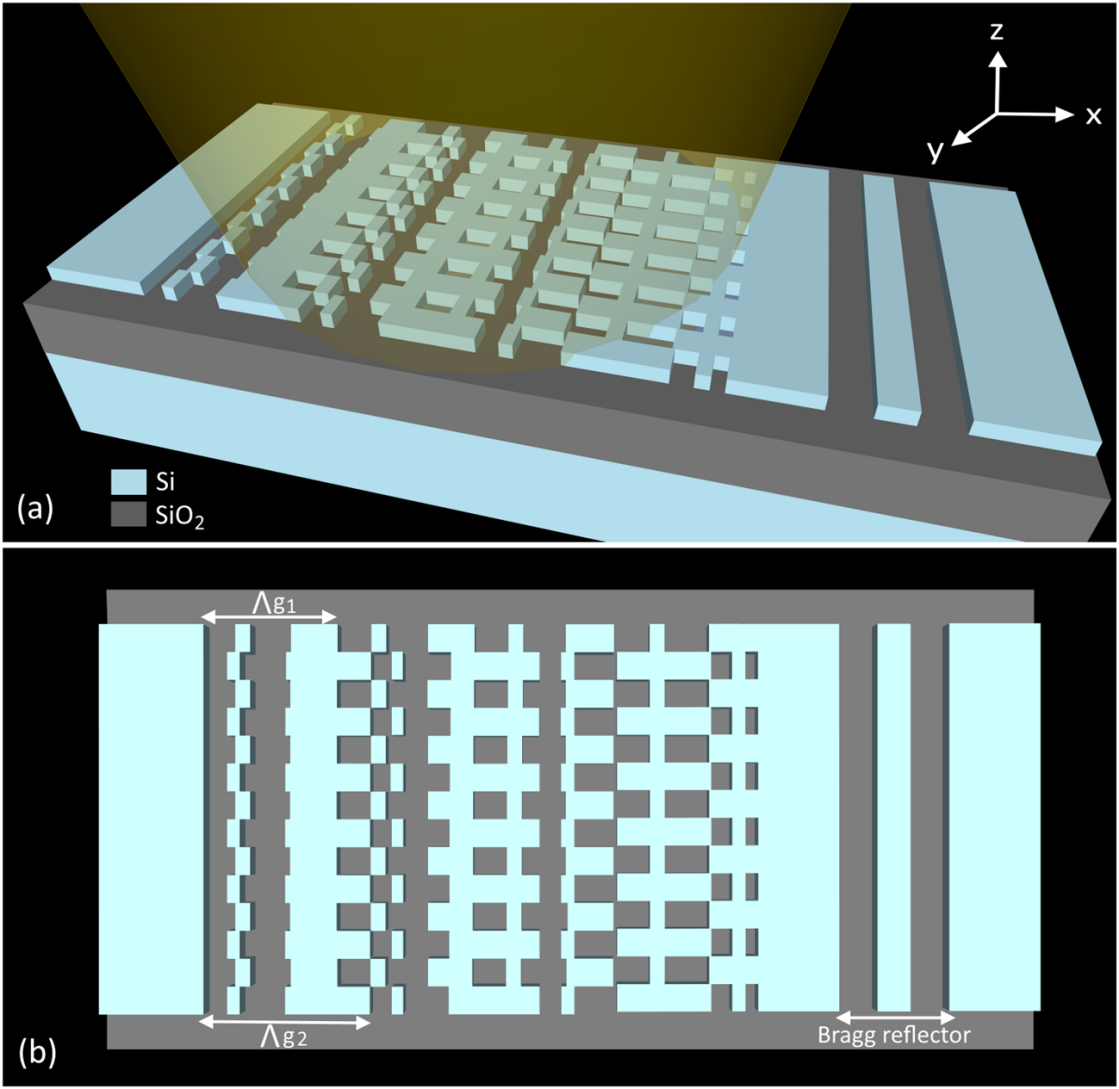
(**a**) Transversally interleaved antenna schematics. The incident light propagates along the x direction, being diffracted of-chip by the antenna nanostructure. The residual power propagating in the waveguide plane is re-injected to the antenna by a terminal Bragg reflector. (**b**) The antenna top view (x–y plane).

Instead of chirping the grating period along the propagation direction, the antenna is implemented by transversally interleaving two gratings with different longitudinal periods, as schematically shown in Fig. 3. Unlike traditional chirped grating, transverse chirping allows for the different chirp periods to equally contribute to the scattered field because the scatterers are arranged in parallel rather than sequentially. We used two of the optimized grating designs described in the longitudinally-chirped antenna section, the first with diffraction angle of − 21° (longitudinal period 600 nm) and the second with diffraction angle of 7° (longitudinal period 733 nm). The two gratings are transversally interleaved fourteen times with a subwavelength pitch of 125 nm, resulting in a total antenna width of 1.75 μm. Each grating has four periods along the longitudinal direction. We find that transversal chirping increases the far-field beam width in the longitudinal direction (as estimated by 3D FDTD simulations) to 43°, an increase of 7° compared to the reference grating described in the periodic grating antenna section.

At the end of the grating, there is part of the light that is left un-diffracted. Adding a Bragg reflector at the end of the grating will further improve grating efficiency by re-circulating the remaining power into the antenna. The reflector comprises two periods of silicon segment with a width of 148 nm separated by 152 nm gaps. The calculated Bragg mirror reflectivity is 95% at a wavelength of 1550 nm. The distance from the reflector to the antenna is judiciously optimized to obtain constructive interference between the forward propagating mode diffracted by the grating and the backward propagating mode diffracted upon reflection. The maximum upward diffraction efficiency of 51% is obtained for a reflector-grating separation of 300 nm, while only 5% of the power remains un-diffracted after the first pass through the antenna and the Bragg mirror. The calculated efficiency is comparable with that of the reference periodic grating, showing that we almost fully compensate the weakening of the grating strengths due to the chirping effect. Furthermore, the use of the Bragg reflector allows to further widen the far-field beam width in the longitudinal direction, as the result of the reversal of the diffraction angle. A gain of an additional 8° is observed in longitudinal beam width, yielding a wide far-field beam of 51° × 47° centered near −12°. In total, the beam width in the longitudinal direction is about 15° larger than the reference case while in transversal direction remains approximately unchanged.

It is important to note that the increased beam divergence is obtained despite the aperture of the interleaved antenna (3 μm × 1.75 μm) being larger than that of the reference design (2.2 μm × 1.75 μm). This confirms that it is possible to circumvent the limitations on far-field beam size for a given antenna aperture by effectively engineering the near-field phase profile of the diffracted light.

Since a relatively large fraction of light (~ 30%) is diffracted downward, this light can be re-directed upward to further enhance the efficiency of the antenna. This is achieved by using a bottom reflector based on a double-SOI substrate, similar to that presented in^37^. The bottom Bragg reflector is designed by optimizing the thickness of the different layers in the double SOI structure. With a silicon thickness of 110 nm sandwiched between two silicon dioxide layers with thicknesses of 500 nm and 274 nm, a reflectivity of about 71% is obtained at the central wavelength of 1550 nm. By incorporating the bottom mirror in our antenna, a diffraction efficiency of 82% is obtained, the downward diffracted power is reduced to 8%, and the back-reflectivity of the antenna is -16 dB.

**Figure 4:**
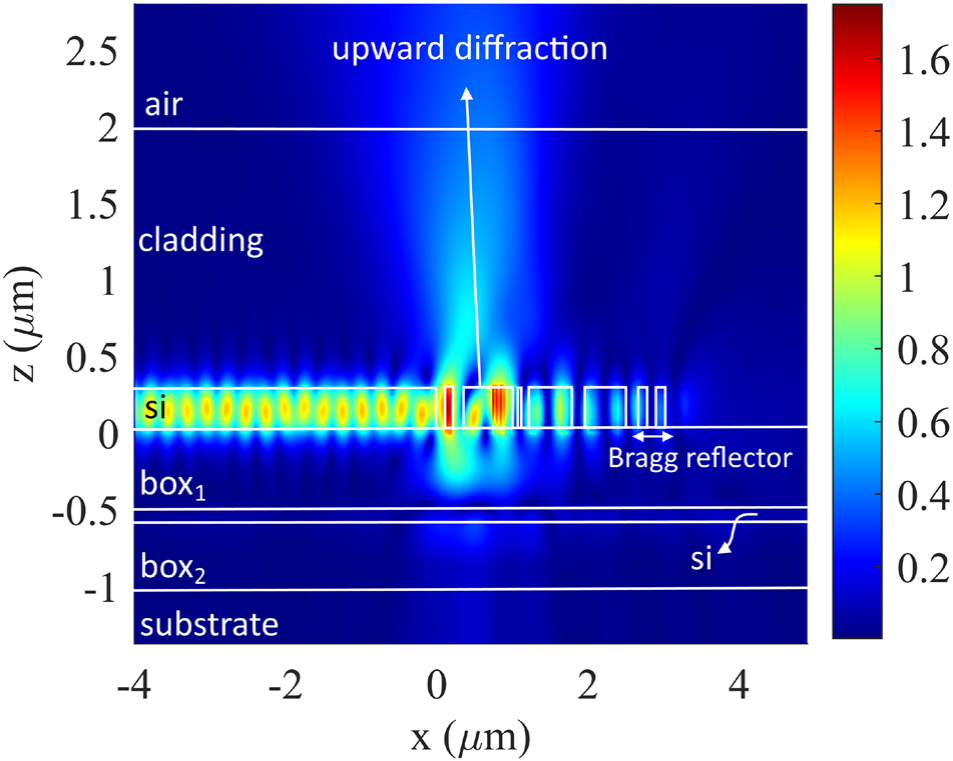
3D FDTD simulation of the amplitude of the scattered electric field on the x–z cross-section positioned at the center of the transversally-interleaved chirped antenna.

**Figure 5:**
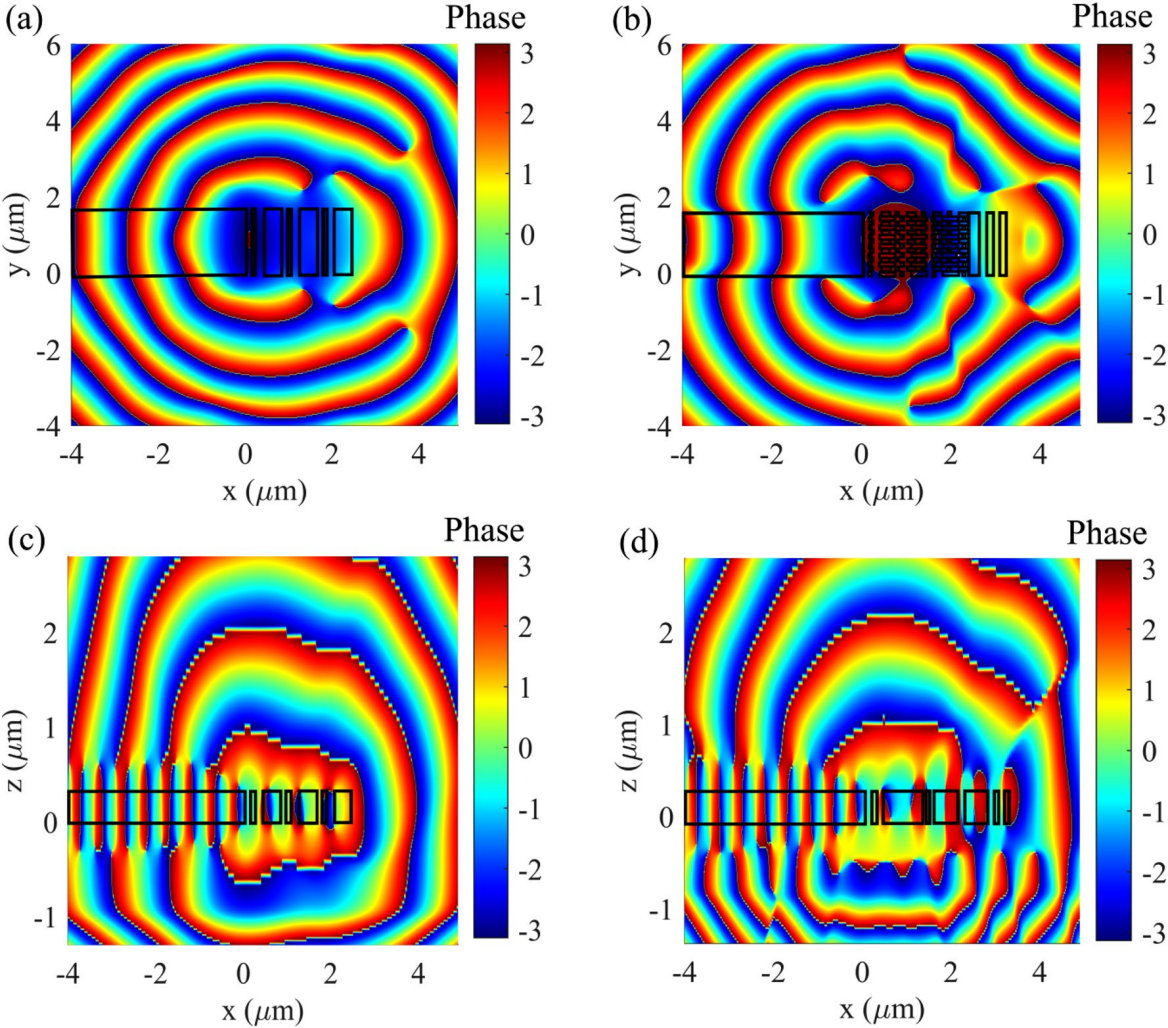
Near-field phases of the electric field on the x–y plane positioned 0.7 μm above the antenna for (**a**) the reference periodic grating and (**b**) the transversally-interleaved chirped antenna at the wavelength of 1550 nm. (**c**) and (**d**) are the phase profiles for the reference periodic grating and the transversally-interleaved chirped antenna, respectively, along the x–z cross-section positioned at the center of the transversally-interleaved chirped antenna as in Fig. 4.

A detailed analysis of the behavior of the designed antenna is reported in Figs. 4 and 5. Figure 4 shows the amplitude of the electric field along the x–z cross-section taken at the center of the antenna. Power is primarily diffracted upward with a peak diffraction angle of –13°. Moreover, there is almost no power remaining in the waveguide after the Bragg reflector. Figure 5 illustrates the phase profiles of the scattered electric field for both the transversally-interleaved chirped antenna (b, d) and the periodic antenna (a, c) along the same x–z cross-section of the Fig. 4 and on a x–y plane placed 0.7 μm above the antenna. The curved phase front induced by the transversally-interleaved chirping can be clearly observed.

**Figure 6:**
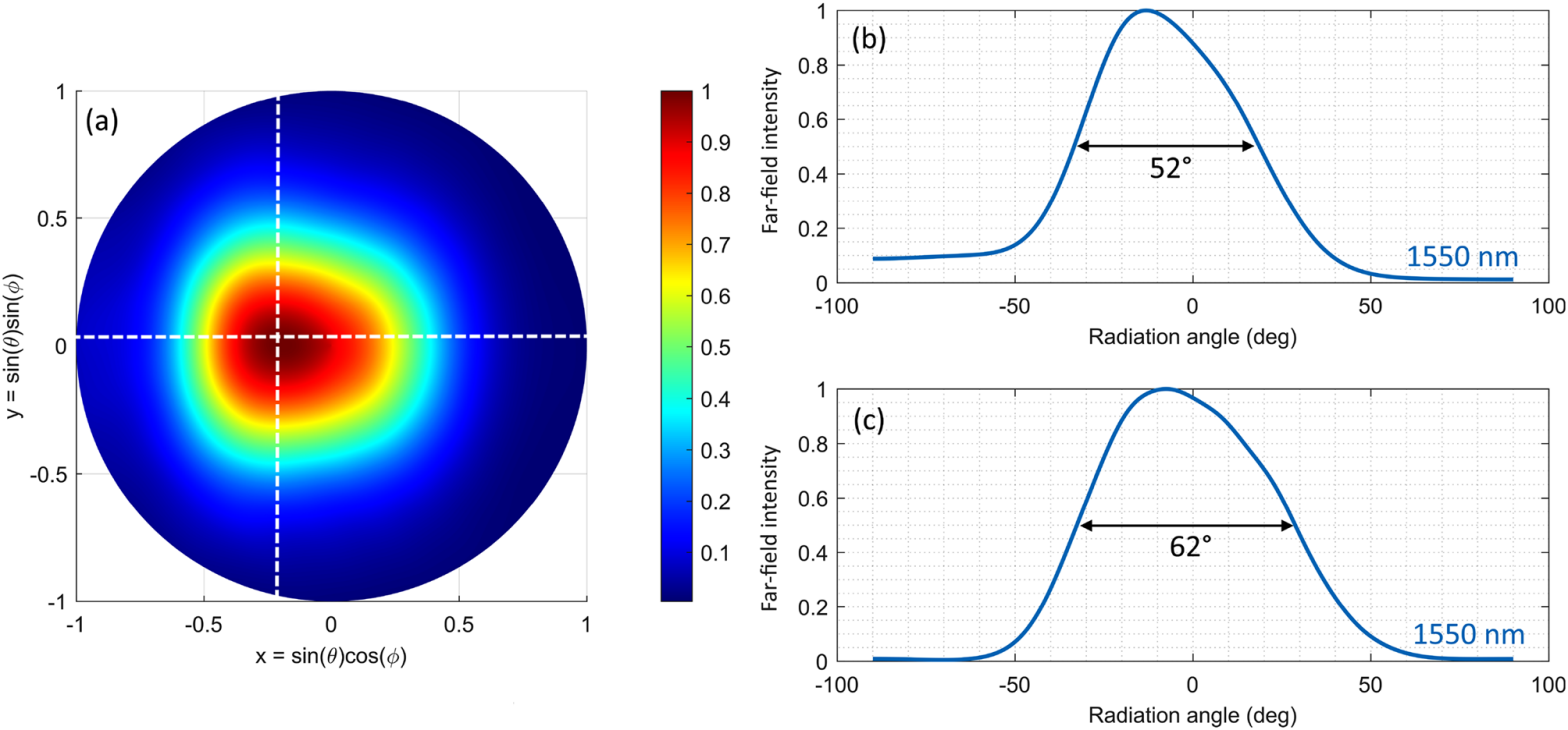
(**a**) The far-field intensity of the optimized antenna design at 1550 nm wavelength. (**b**) Far-field intensity distribution along the x and (**c**) y axis, as marked by the dashed white lines in (**a**).

Figure 6 shows the simulated far-field of the designed antenna. As can be seen, the additional phase curvature in the near-field obtained through transversal chirping translates into a wide far-field beam width of 52° × 62°. The peak diffraction angle is at -13° as expected from the near-field analysis.

Lastly, we performed a tolerance analysis to investigate the sensitivity of the optimized antenna design to fabrication imperfections. In particular, we evaluated the change in diffraction efficiency for segment length variations. The antenna shows a robust performance, i.e. less than a 6% reduction in diffraction efficiency, for fabrication bias of ± 5 nm, which is achievable using state-of-the-art silicon photonics manufacturing^38^.

## Discussion and conclusion

In this paper, we demonstrate a new strategy based on near-field phase engineering to circumvent fundamental limitations on the beam width that can be achieved in optical antennas. Phase engineering is realized using transversally-interleaved subwavelength grating nanostructures followed by a Bragg reflector. Our optimized antenna design achieves a FWHM of 52° × 62°, a high diffraction efficiency of 82% (exploiting a bottom reflector), and an ultra-compact footprint of 3.15 μm × 1.75 μm. To compare our antenna with the state-of-the-art designs, we note that in ref. ^39^ a dual-input antenna based on two waveguides placed on opposite sides of a surface grating was used to increase the beam width. While this approach implements a concept similar to the use of a Bragg reflector that we propose here, it adds the additional burden of having two inputs with a perfectly controlled phases to properly work. Moreover, the angular range of the aforementioned design is limited to 40° × 15° for an effective antenna aperture of 2.5 μm^2^, much smaller compared to our design result reported here. This confirms once more the effectiveness of our transverse chirping strategy in substantially broadening the antenna far-field. We expect that exploration of near-field phase engineered nanostructures and integrated photonic components using transverse interleaved subwavelength gratings will lead to a new research direction in the development of integrated photonics antennas for a wide range of applications, including free-space optical interconnects and on-chip optical phased arrays for lidar systems.

## Data Availability

Data generated or analyzed in this study may be obtained from the contact author upon reasonable request.
